# Comparison of Multivariate Regression Models Based on Water- and Carbohydrate-Related Spectral Regions in the Near-Infrared for Aqueous Solutions of Glucose

**DOI:** 10.3390/molecules24203696

**Published:** 2019-10-15

**Authors:** Anel Beganović, Vanessa Moll, Christian W. Huck

**Affiliations:** Institute of Analytical Chemistry and Radiochemistry, CCB-Center for Chemistry and Biomedicine, Innrain 80/82, 6020 Innsbruck, Austria; anel.beganovic@uibk.ac.at (A.B.); vanessa.moll@student.uibk.ac.at (V.M.)

**Keywords:** FT-NIR spectroscopy, PLS-R, water, glucose, test set validation, RMSEP

## Abstract

The predictive power of the two major water bands centered at 6900 cm${}^{-1}$ and 5200 cm${}^{-1}$ in the near-infrared (NIR) region was compared to carbohydrate-related spectral areas located in the first overtone (around 6000 cm${}^{-1}$) and combination (around 4500 cm${}^{-1}$) region using glucose in aqueous solutions as a model substance. For the purpose of optimal coverage of stronger as well as weaker absorbing NIR regions, cells with three different declared optical pathlengths were employed. The sample set consisted of multiple separately prepared batches in the range of 50–200 mmol/L. Moreover, the samples were divided into a calibration set for the construction of the partial least squares regression (PLS-R) models and a test set for the validation process with independent samples. The first overtone and combination region showed relative prediction errors between 0.4–1.6% with only one PLS-R factor required. On the other hand, the errors for the water bands were found between 1.6–8.3% and up to three PLS-R factors required. The best PLS-R models resulted from the cell with 1 mm optical pathlength. In general, the results suggested that the carbohydrate-related regions in the first overtone and combination region should be preferred over the regions of the two dominant water bands.

## 1. Introduction

Glucose is of great importance in physiological systems, medicine, and health care, as well as in the food and beverage industry. Besides other analytical methods, glucose and other carbohydrates are often quantitatively determined enzymatically or via chromatographic methods such as high performance liquid chromatography (HPLC) or gas chromatography (GC) [1,2,3]. However, these methods are rather time-consuming and expensive as they usually require sample preparation, long measurement time (incubation of enzymes, separation on column, etc.), and qualified personnel. Considering these drawbacks of conventional analytical techniques, near-infrared spectroscopy (NIRS) is of increasing interest as an alternative method for the quantification of glucose and other carbohydrates in aqueous solutions. NIRS mostly does not require any sample preparation, offers fast and non-invasive analyses, and multiple sample characteristics are accessible with one single measurement. Moreover, NIR spectrometers are cheap to run and can be operated by relatively untrained personnel. Alongside the mentioned advantages, NIRS comes with a few downsides. One is that the information contained in NIR spectra often needs to be extracted using multivariate data analysis tools such as principal component analysis (PCA) or partial least squares regression (PLS-R). The other is, in order to establish a robust model for a reliable prediction of future samples, sufficient reference measurements with known target parameters need to be provided to the calibration. Both mentioned drawbacks make NIRS time-consuming and require highly skilled personnel in the calibration phase, and therefore might be cost-intensive initially [4,5,6].

Glucose in water and in various other water-based matrices like buffer and serum solutions, blood anticoagulants, fruit juices, and alcoholic beverages was extensively studied in the field of NIRS. Furthermore, non-invasive blood glucose monitoring and glucose quantifications in other body fluids (e.g., urine) using NIRS is a matter of great interest in literature. The aqueous NIR spectrum of glucose is dominated by intense water bands centered at around 6900 cm${}^{-1}$ and 5200 cm${}^{-1}$, which are assigned to combinations of water OH stretching and bending modes [7,8,9,10]. Depending on the utilized optical cell pathlength (distance of interaction between sample and light), these bands can become unusable due to the complete absorption of NIR radiation by water [11,12,13]. Clearly visible glucose-related bands usually do not appear at lower concentrations in the NIR spectrum of aqueous glucose without spectral pre-treatments (e.g., derivative functions) [14], but become apparent at higher concentrations [15]. However, prominent absorption features of dissolved carbohydrates are located in the combination region around 4500 cm${}^{-1}$, the first overtone region around 6000 cm${}^{-1}$, as well as in the short wavelength NIR region around 9500 cm${}^{-1}$ [16,17,18]. In case of carbohydrates, vibrations in these regions are mostly due to C−H-based combination and overtone vibrations [8,19]. The available literature is primarily concerned with the combination and first overtone region.

Addition of glucose or other carbohydrates may have a similar effect on the liquid water structure (hydrogen bond network) as an addition of inorganic salts [20,21,22] or changes in temperature [10,23,24]. Thereby, changes in the appearance of the water bands in the NIR region are induced [13,25,26,27]. Furthermore, carbohydrates in aqueous solution can act on the water cluster as structure breakers or structure makers [26]. The extent of the changes in the water structure is connected to the concentration of the corresponding solute and thus the water bands can be utilized for quantitative analyses [13,25,27]. As already mentioned, glucose and other carbohydrates exhibit characteristic NIR spectral features in the combination, first overtone, and short wavelength NIR region, next to the two dominant water bands. The direct relation between the concentration of dissolved carbohydrates and the spectral response allowed successful applications of these regions in previous quantification studies [14,16,17,18,28,29].

Chen et al. [17] compared the predictive power of the combination and first overtone region for glucose and other biomolecules in aqueous solutions in the range of approximately 0–35 mM. The authors optimized the optical pathlength for each of the two regions and concluded that the combination region was superior relative to the first overtone NIR region. Beganović et al. [13] investigated the performance of the two major water bands in the NIR centered at around 6900 cm${}^{-1}$ and 5200 cm${}^{-1}$. In order to overcome the issue of complete absorption of NIR light most commonly occuring at the water band located around 5200 cm${}^{-1}$, they utilized a cell with 0.1 mm optical pathlength. By this, the authors demonstrated rich information content of the so-called combination band of water at 5200 cm${}^{-1}$, which is often not taken into consideration in literature. Compared to the water band at 6900 cm${}^{-1}$, the authors reported lower prediction errors for the more intense water band at 5200 cm${}^{-1}$. To best of our knowledge, no previously conducted study compared the performance of all these NIR regions directly—the water-based, as well as the carbohydrate-based regions. The present study intends to point out the NIR regions with the maximum of relevant information content for the analysis of carbohydrate-based aqueous solutions. This is of particular interest for applications working close to the limits of detection (LOD) as well as limits of quantification (LOQ).

Therefore, this work aims at the comparison of the two dominating waterbands in the NIR centered at around 6900 cm${}^{-1}$ and 5200 cm${}^{-1}$ to the sugar-related spectral regions located in the first overtone and combination region around 6000 cm${}^{-1}$ and 4500 cm${}^{-1}$, respectively. Glucose in aqueous solutions is used as a model substance. In addition, cells with different optical pathlengths are utilized in order to access the whole NIR region from 10,000–4000 cm${}^{-1}$ (thinner cell pathlength) and to account for the lower absorption in the first overtone region (thicker cell pathlength).

## 2. Material and Methods

### 2.1. Samples

d-(+)-glucose (≥99.5%) was purchased from Carl Roth (Karlsruhe, Germany) and Milli-Q water with a resistivity of 18.2 MΩ cm was used for the preparation of the glucose solutions. The calibration set was composed of pure Milli-Q water and glucose concentrations ranging from 50–200 mmol/L in steps of 30 mmol/L. For the test set, samples with glucose concentrations of 60.2, 130.5 and 186.0 mmol/L were prepared.

In order to avoid any effects of measurement time, multiple independent batches for both calibration and test samples were prepared. The calibration and test set consisted of three and two batches per sample, respectively. The preparation and measurement of the samples were randomized. Furthermore, all samples were measured on the day they were prepared.

### 2.2. FT-NIR Measurements

The Büchi NIRFlex N-500 FT-NIR spectrometer (Büchi, Flawil, Switzerland) equipped with the liquids measurement cell was used to acquire NIR spectra of the glucose solutions. The spectra were recorded in transmission mode in the range of 10,000–4000 cm${}^{-1}$ with a spectral resolution of 8 cm${}^{-1}$, while each sample was scanned 64 times. In order to account for the lower absorption towards increasing wavenumbers, the measurements were performed using three cell types with different declared optical pathlengths. The cells were purchased from Hellma GmbH & Co. KG (Müllheim, Germany) and specified as follows: one 106-QS quartz SUPRASIL^®^ cell with 0.1 mm optical pathlength and demountable cell windows, and multiple 100-QX quartz SUPRASIL^®^ cells with 1 mm and 2 mm optical pathlength, respectively. Spectrometer reference measurements were performed in the beginning, as well as in the middle of each measurement day. Data acquisition was accomplished using the NIRWare 1.4.3010 software package (Büchi, Flawil, Switzerland).

Samples were always freshly prepared and measured randomly over a period of four weeks. In contrast to the 1 mm and 2 mm cells—which were simply filled with a certain amount of sample solution—the 0.1 mm cell is demountable and thus had to be filled differently. For the 0.1 mm cell, approximately 40 μL of glucose solution was applied onto the sample recess of one optical cell window, followed by the careful attachment of the second cell window. Excessive sample solution was displaced and collected with a tissue. Sticky glucose residues on the outside surface of the cell were removed.

The NIR measurements were performed at 35 due to the fact that the NIRFlex N-500 liquids measurement cell was subjected to significant fluctuations at lower temperatures. However, at 35 , the temperature fluctuation stabilized at ±0.1 [13]. In order to avoid the introduction of temperature-driven shifts in the NIR spectrum, each cell filled with sample solution was tempered to 35 before the measurements were started. The 0.1 mm cell was thermally equilibrated for 30 s, while the 1 mm and the 2 mm cells were thermally equilibrated for 1 min and for 2 min, respectively. To avoid water evaporation, the 1 mm and 2 mm cells were covered with a lid, whereas the 0.1 mm cell did not offer any cover possibility since the two cell windows were kept together by adhesion. The only possibility to prevent the evaporation of water out of the 0.1 mm cell was to minimize the time between the filling of the cell with sample solution and the actual sample measurement.

All samples were measured nine times, while the cells were refilled with fresh solution for each of the nine repeat measurements. The cells were cleaned thoroughly after every single measurement using Milli-Q water and ethanol. Lint-free tissues, as well as a conventional compressed air system, were used in order to dry the cells and remove potential dust particles.

### 2.3. Band Assignment and Division of Spectral Regions

In this study, the two water bands at around 6900 cm${}^{-1}$ and 5200 cm${}^{-1}$, as well as two regions of glucose-related vibrations located at around 5900 cm${}^{-1}$ and 4400 cm${}^{-1}$, were used for the comparison of the predictive power of each region separately at different cell pathlengths. Since water is known to be a strong absorber in the near-infrared [8,30], complete absorption of the NIR light can occur in certain spectral regions—depending on the cell pathlength, infrared source, and detector [30]. As a consequence, the water band centered at 5200 cm${}^{-1}$ could not be utilized for the purpose of any quantitative analysis using both the 1 mm and 2 mm cells. This region was only accessible using the 0.1 mm cell.

The NIR regions related to water were selected in such a way that they ranged from the beginning to the end of the corresponding NIR band while the regions related to glucose were chosen according to the spectral pattern after the application of the second derivative discussed later (Section 3). For the water band at around 6900 cm${}^{-1}$ a spectral range of 7692–6248 cm${}^{-1}$ was selected in order to match the region frequently used in aquaphotomics [27], and was labeled as W1 in this study. This band is commonly referred to as the first overtone of water [27,31], although it is actually a combination of symmetric and antisymmetric stretching vibration modes of water [8,9]. The spectral region for the second water band—labeled as W2—was set to 5400–4600 cm${}^{-1}$ and is assigned to the combination of bending and antisymmetric water stretching modes [7,8].

**Figure 1:**
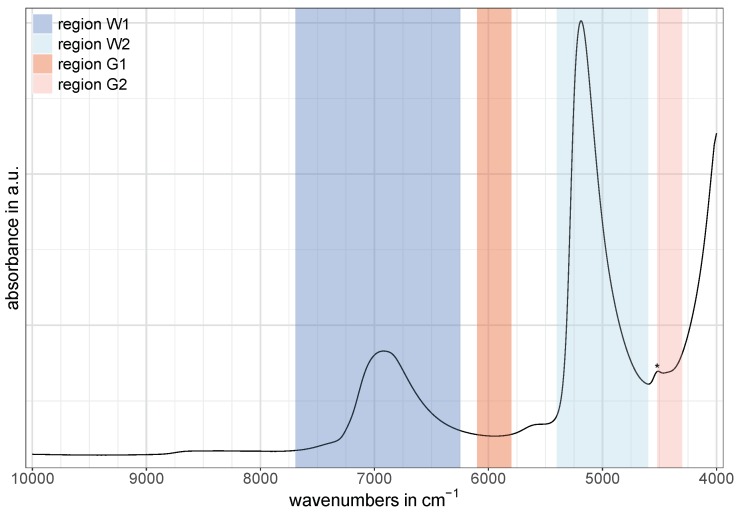
Illustration of the division of the spectral regions W1, W2, G1, and G2 using an exemplary spectrum collected with the 0.1 mm cell. The O−H residue in the cell’s quartz windows at around 4500 cm${}^{-1}$ is marked with an asterisk. It is assigned to a combination of an O−H stretching vibration and one of the SiO_2_ fundamental vibrations [8,34].

The two regions at around 5900 cm${}^{-1}$ and 4400 cm${}^{-1}$ related to glucose vibrations in water were labeled as G1 and G2, respectively. For G1, the spectral region was set to 6100–5800 cm${}^{-1}$ and is assigned to first overtone vibrations of C−H compounds [8,16,32]. The spectral region for G2 was set to 4520–4300 cm${}^{-1}$ and is assigned to combinations of C−H stretching and CH_2_ deformation vibrations, as well as combinations of stretching vibrations of glucose-related O−H and C−O compounds [8,16]. Figure 1 shows an exemplary NIR spectrum of water containing glucose with the described division of spectral regions. Note that the small band around 4500 cm${}^{-1}$ (marked with an asterisk in Figure 1) is caused by O−H residues in the quartz windows of the 0.1 mm cell (probably due to water impurities [33]) and is assigned to a combination of an O−H stretching vibration and one of the SiO_2_ fundamental vibrations [8,34].

### 2.4. Multivariate Data Analysis

The Unscrambler X Ver. 10.5 (Camo Software AS, Oslo, Norway) was used for the pre-treatment of the NIR spectra as well as the construction and validation of the multivariate regression models. Due to the occurrence of interference fringes using the 0.1 mm cell, the frequency filtering technique fast Fourier transform filter (FFT-filter) [13] was applied to these NIR spectra using OriginPro Ver. 9.1G (OriginLab Corporation, Northampton, MA, USA). Thereby, the NIR spectra were first Fourier transformed, followed by the application of a filter function and finally retransformed by inverse Fourier transformation. In order to only eliminate the disturbing interferences and leave the regular spectra containing the targeted information untouched, the parabolic low-pass filter was chosen as an FFT filter function. This filter blocks all frequencies above a certain threshold value (cutoff frequency), while lower frequency elements are allowed to pass [13]. The cutoff frequency was set to 0.02625 Hz—all frequencies above were eliminated before the spectra were inverse Fourier transformed. The suitability of this approach has been validated before [13]. Afterward, the spectra of all three cell pathlengths were transformed from transmittance to absorbance.

The NIR spectra were reduced batchwise from nine spectra to one representative spectrum for each batch. All previously defined spectral regions were subjected to an individual optimization of pre-treatments (see Table 1). However, in case of the regions W1 and W2, the pre-treatments were chosen as proposed in aquaphotomics literature [27] with an additional application of a standard normal variate (SNV) transformation [35]. For the regions G1 and G2, it was found that a second order Savitzky–Golay derivative [36] with a second order polynomial and a varying number of smoothing points was optimal. Second order derivative spectra were also calculated for the two water-related regions W1 and W2. The results were inferior compared to the pre-treatments mentioned above and will therefore not be discussed any further.

For each spectral region, regression models were calculated using partial least squares regression (PLS-R) along with the NIPALS algorithm. The calibration process incorporated 21 calibration samples from three batches, whereas the performance of the calibration models was evaluated with six completely independent samples from two batches (test set validation). Note that these samples were never employed in any calibration [37,38]. The performances of the PLS-R models were assessed using the root mean square error (RMSE), which was calculated according to Equation (1), where $y_{i}$ and ${\hat{y}}_{i}$ represent the reference and predicted values, respectively. Furthermore, in order to enable a more straightforward interpretation of the RMSE’s scale, a percentage error called normalized RMSE (NRMSE) was introduced, which refers to the calibration range of 0–200 mmol/L (see Equation (2)). The errors of the calibration (CAL) and test set validation (TSV) were referred to as root mean square error of calibration (RMSEC) and root mean square error of prediction (RMSEP), respectively:

(1) $$RMSE=\sqrt{\frac{1}{n}\sum_{i=1}^{n}{\left(y_{i}-{\hat{y}}_{i}\right)}^{2}},$$

(2) $$NRMSE=\frac{RMSE}{y_{max}-y_{min}}\times 100.$$

The RMSE’s magnitude is closely associated with the number of PLS-R factors (or latent variables), which is a crucial parameter for a satisfactory performing PLS-R model [37,38]. Since the glucose-water system used in this study is rather simple, the number of PLS-R factors employed in the PLS-R models should be kept quite low in order to avoid modeling of noise and thus non-relevant spectral information (overfitting). However, using too few PLS-R factors can lead to poor model performance due to the lack of explained variance in the NIR spectra (underfitting). The optimal number of PLS-R factors was determined by the examination of the regression coefficients, the loadings and correlation loadings of each PLS-R factor as well as the explained variances.

## 3. Results and Discussion

**Figure 2:**
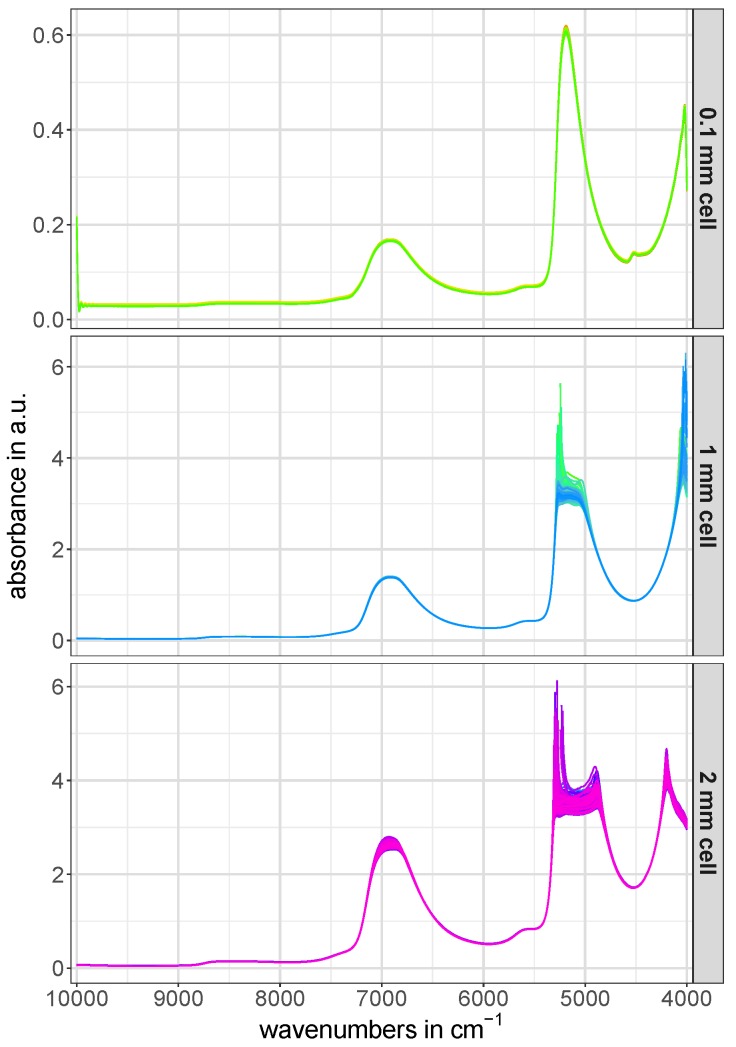
Raw NIR spectra (calibration and test set) of all three utilized cell pathlengths. The spectra were only transformed from transmittance to absorbance.

The full-range raw NIR spectra of the calibration and test set for all three utilized cell pathlengths are depicted in Figure 2. The artifacts occuring in the region around 5200 cm${}^{-1}$ in the raw NIR spectra of the 1 mm and 2 mm cells in Figure 2 are caused by the complete absorption of NIR light in this spectral region [11].

**Figure 3:**
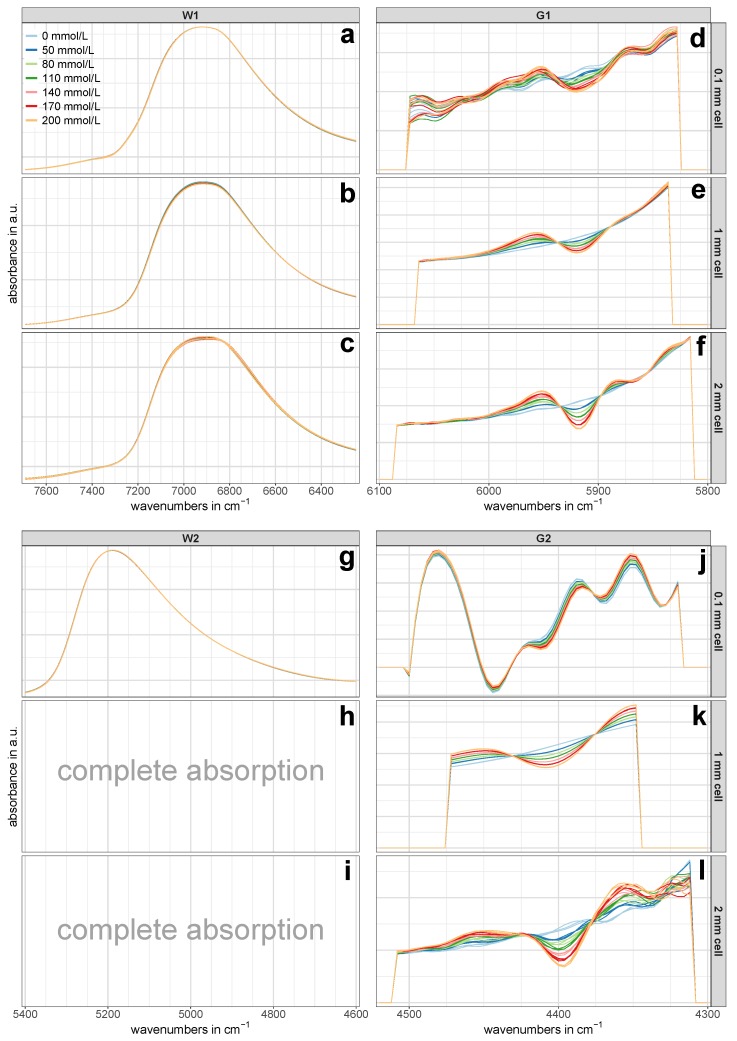
Pre-treated NIR spectra of the calibration set. The spectral regions W1 (**a**–**c**), W2 (**g**), G1 (**d**–**f**), and G2 (**j**–**l**) are shown for all three utilized cell pathlengths separately. Each concentration is represented by three representative NIR spectra (one per batch). No spectra were available in region W2 for the 1 mm and 2 mm cells (**h**,**i**) due to the complete absorption of the NIR light.

**Figure 4:**
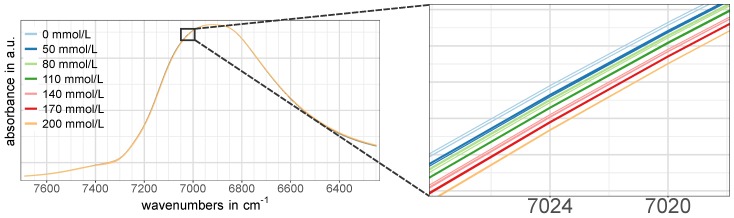
Illustration of the glucose concentration dependent pattern in the water band located at around 6900 cm${}^{-1}$ (region W1). NIR spectra of the calibration set recorded with the 0.1 mm cell are shown here exemplarily.

The pre-treated NIR calibration set spectra of the regions W1, W2, G1 and G2 for all three cell pathlengths are presented in Figure 3. Since each of the three batches per concentration was averaged from nine spectra to one representative spectrum, three spectra per concentration are shown in Figure 3. The glucose-related regions G1 and G2 showed an evident pattern towards increasing glucose concentrations (Figure 3e,f,j–l), while such an obvious pattern was missing in the NIR spectra of the water-related regions W1 and W2 at first glance (Figure 3a–c,g). However, a closer look revealed that there actually was a certain concentration dependent pattern, although it was not as pronounced as in the regions associated with glucose vibrations. An example of this is shown in Figure 4.

In the course of data analysis, the number of smoothing points for the second derivative in the first overtone and combination region was individually optimized prior to the PLS-R. As a consequence, the exact spectral range used for the PLS-R varied for each cell. Nevertheless, the spectral regions subjected to the calculation of the derivative spectra did not vary in between the three cell pathlengths.

### 3.1. Measurements with 0.1 mm Cell Pathlength

**Figure 5:**
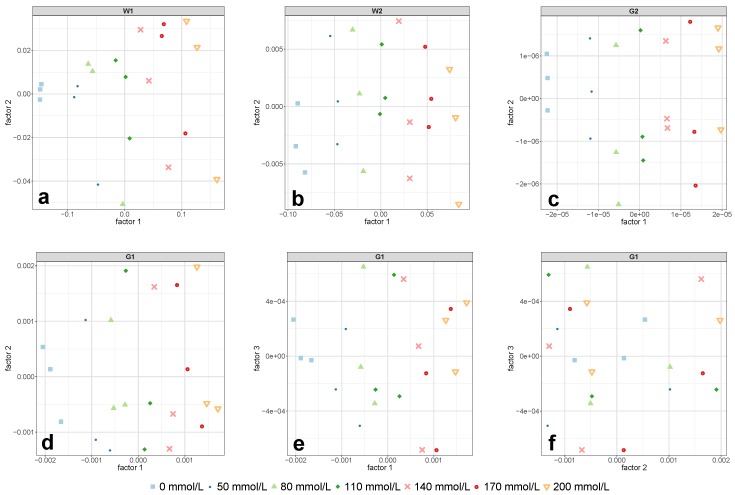
PLS-R score plots of the calibration set for the regions W1 (**a**), W2 (**b**), G2 (**c**), and G1 (**d**–**f**) obtained by measurements with the 0.1 mm cell. Each data point corresponds to one batch. The featured factors were chosen according to the required number of PLS-R factors in Table 2.

The results of the PLS-R calibration and test set validation procedure is presented in Table 2. The 0.1 mm cell allowed the evaluation of the performance of all four investigated regions. Comparing the results for the 0.1 mm cell in Table 2, probably the most noticeable value is the relatively high prediction error of RMSEP = 22.6 mmol/L of the first overtone region G1. This error’s magnitude of more than 11% employing three PLS-R factors was hardly surprising, considering the lack of a distinct concentration dependent pattern in Figure 3d. The reason for this was that a small amount of interference fringes was still present in this spectral region and that there was insufficient spectral information content due to the short pathlength [30]. These remaining fringes were hardly noticeable in the regular absorption spectrum but became evident after the application of the second derivative—despite previous smoothing of the NIR spectra. Considering the PLS-R scores of the calibration set of region G1 in Figure 5d–f, the first PLS-R factor mostly accounted for the changes in glucose concentration. Despite that, the PLS-R calibration model was not able to predict the test set adequately.

**Figure 6:**
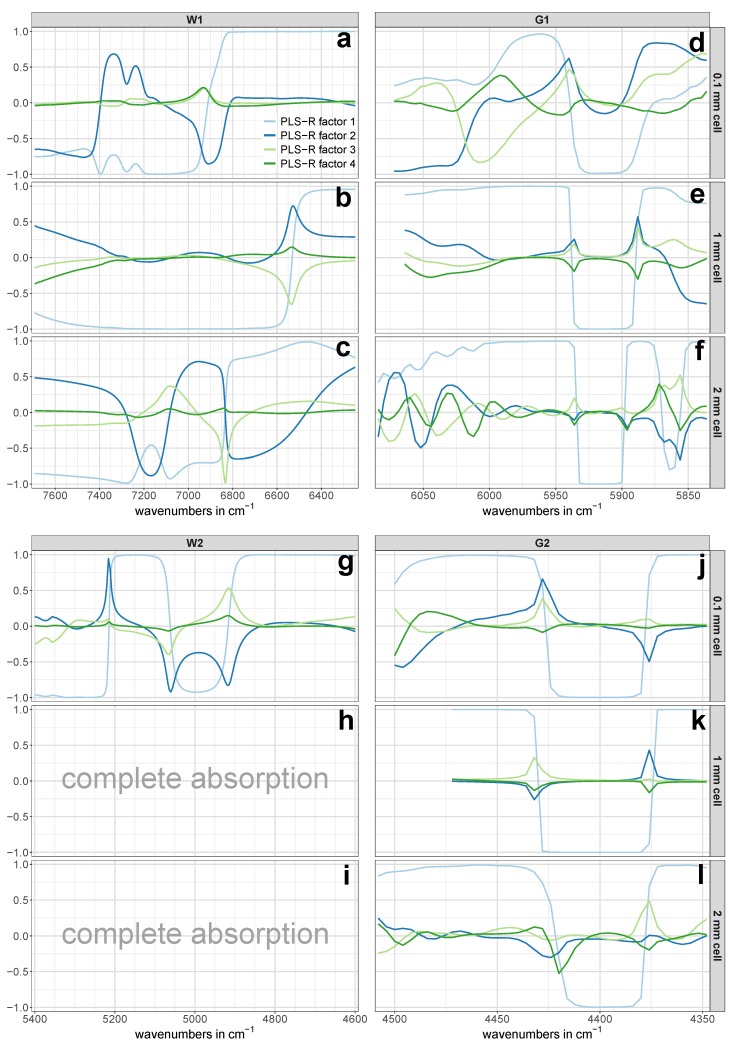
Correlation loadings of the first four PLS-R factors for the regions W1 (**a**–**c**), W2 (**g**–**i**), G1 (**d**–**f**), and G2 (**j**–**l**) with the corresponding cell pathlengths.

The models for the two water-related regions W1 and W2 both showed similar percentage errors of around NRMSEP = 4% for the prediction of unknown samples from the test set using two PLS-R factors, respectively. A consideration of more than two PLS-R factors for each model would have further reduced the prediction error; however, a closer look at the model statistics gave no justification for the use of a third PLS-R factor. In case of region W1, the validation model’s explained Y-variance (variance in glucose concentration) comparably increased from PLS-R factor 1 to 2 and from PLS-R factor 2 to 3 (see Table 3), and therefore suggested a model based on three PLS-R factors. In contrast, the correlation loadings of PLS-R factor 3 showed very low values with a maximum of 0.2 (see Figure 6a), which led to the exclusion of PLS-R factor 3 from the PLS-R model due to the risk of modeling glucose-unrelated spectral information. For the combination band of water (region W2), two PLS-R factors were considered as optimal as the explained Y-variance in the PLS-R validation model increased by 2.4% from PLS-R factor 1 to 2 (see Table 3) and the correlation loadings indicated many X-variables with strong contributions to the second PLS-R factor (see Figure 6g).

Among all prediction errors of the measurements conducted with the 0.1 mm cell, the glucose-related combination region G2 yielded by far the lowest prediction error. The model required only one PLS-R factor to yield an NRMSEP as low as 0.7% along with an R${}_{\mathrm{TSV}}^{2}$ of 0.9993. The explained Y-variance of the test set already reached 99.9% in the first PLS-R factor (see Table 3), and thus made the use of more PLS-R factors invalid. This remarkable prediction performance of region G2 can be attributed to the very distinct concentration pattern in the second derivative NIR spectra (see Figure 3j), which was not observed in the regular (untreated) spectra. In addition to that, the concentrations in the PLS-R score plot of region G2 in Figure 5c were perfectly separated along PLS-R factor 1. This demonstrated that PLS-R factor 1 exclusively accounted for changes in glucose concentration and thus allowed the exclusion of further PLS-R factors.

### 3.2. Measurements with 1 mm Cell Pathlength

The performance of the three exploitable regions of the 1 mm cell was remarkable. The PLS-R calibration model of the so-called first overtone of water (region W1) predicted the independent test set samples with an error of RMSEP = 3.2 mmol/L and a relative error of NRMSEP = 1.6% (see Table 2). These errors were achieved using the first two PLS-R factors and together accounted for 99.7% of the Y-variance (see Table 3), which, as a consequence, did not allow the consideration of further PLS-R factors in the model for region W1.

**Figure 7:**
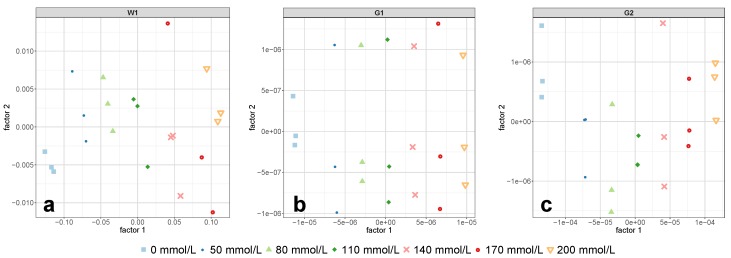
PLS-R score plots of the calibration set for the regions W1 (**a**), G1 (**b**), and G2 (**c**) obtained by measurements with the 1 mm cell. Each data point corresponds to one batch. The featured factors were chosen according to the required number of PLS-R factors in Table 2.

The pre-treated NIR spectra of the glucose-related regions G1 and G2 in Figure 3e,k, respectively, showed the same concentration dependent pattern from pure water towards increasing glucose content. This clearly evident pattern indicated that glucose in aqueous solution produces own NIR bands. This finding is in contrast to the frequently found view in the literature [26,39], according to which carbohydrates do not exhibit own NIR bands in aqueous solutions, but rather characteristically disturbs the water structure. Actually, at low concentrations, these bands are more like tiny changes in the untreated spectra’s path line, which cannot be recognized by the eye, but are rather revealed and highlighted by calculating derivative spectra. The high predictive power of the two glucose-related regions is best represented by the low errors in the prediction of the independent test set: the PLS-R model for the first overtone region G1 yielded an NRMSEP value of 0.9%, whereas the NRMSEP for the combination region G2 was as low as 0.4% (see Table 2). The fact that for each PLS-R model only one PLS-R factor was necessary to achieve the mentioned prediction errors using the two regions associated with glucose vibrations showed the distinct glucose-related nature of these regions. The use of only one PLS-R factor for the two glucose-related regions was further confirmed by the fact that the concentrations in the PLS-R score plots in Figure 7a,b were clearly separated along the first PLS-R factor.

However, these findings allow for reconsidering the statement of Chen et al. [17], according to which a 1 mm cell pathlength is too thin for satisfactory glucose quantification from NIR spectra in the first overtone region in an aqueous matrix. The authors of the aforementioned study did not use derivative spectra. In contrast to Chen et al. [17], the results presented herein rather suggest that a cell pathlength of 1 mm is perfectly suitable. By applying a second derivative function to the first overtone region, a clear concentration dependent pattern becomes evident (see Figure 3e) and thus allows the construction of highly accurate PLS-R models for glucose quantifications. Our study did not investigate the exact cell pathlength at which it becomes too thin for high-quality NIR spectra. Nevertheless, considering the relatively high prediction error of the 0.1 mm cell in region G1, it can be concluded that this limit is below a cell pathlength of 1 mm.

### 3.3. Measurements with 2 mm Cell Pathlength

For the 2 mm cell, the test set validation for the so-called first overtone of water (region W1) yielded an NRMSEP of around 5% utilizing three PLS-R factors (see Table 2). An additional consideration of PLS-R factor 4 would have reduced the relative error by nearly half, but, from the interpretation of the model statistics, it was concluded that this would have led to the modeling of noise or glucose-unrelated spectral information. Although the explained Y-variance of the validation model increased by 2.4% from PLS-R factor 3 to PLS-R factor 4 (see Table 3), the correlation loadings showed negligibly small values for PLS-R factor 4 (see Figure 6c). This suggested that the X-variables modeled in PLS-R factor 4 were not of importance for the regression model and therefore might have contained non-relevant spectral information for the quantification of the target solute.

**Figure 8:**
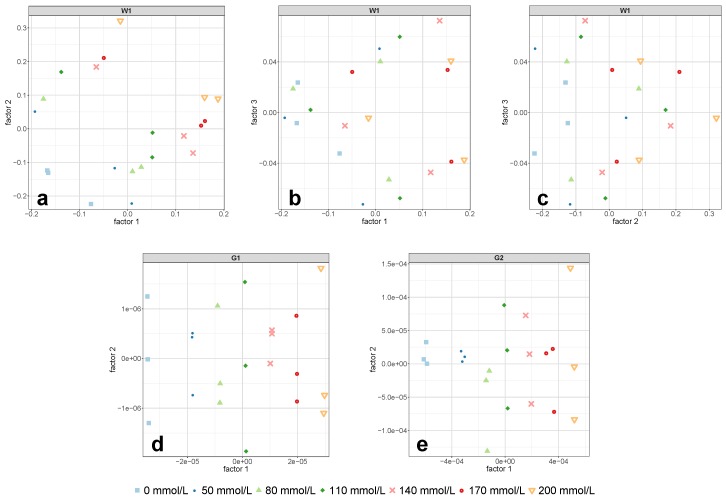
PLS-R score plots of the calibration set for the regions W1 (**a**–**c**), G1 (**d**), and G2 (**e**) obtained by measurements with the 2 mm cell. Each data point corresponds to one batch. The featured factors were chosen according to the required number of PLS-R factors in Table 2.

In direct comparison to the two glucose-related regions, the predictive power of region W1 was inferior. Using a pathlength of 2 mm, the PLS-R calibration models for the regions G1 and G2 predicted the independent test set samples with prediction errors of NRMSEP = 0.8% and NRMSEP = 1.6%, respectively, whereas both models required only one PLS-R factor (see Table 2). The second derivative spectra in Figure 3f,l showed a clear glucose concentration dependent pattern towards increasing glucose content, which was also reflected in the PLS-R score plots in Figure 8d,e. However, compared to the derivative spectra of the 1 mm cell in the combination region G2 (Figure 3k), the spectra in Figure 3l appeared noisy to some extent. This noisy pattern could be removed with a higher number of smoothing points in the second derivative, but the test set validation yielded poorer RMSEP values and required more PLS-R factors. It is conceivable that the high absorption of the adjacent water combination band and the associated spectral artifacts (see Figure 2) had an impact on region G2 and consequently led to the somewhat higher prediction error in this region.

### 3.4. Comparison between Cell Pathlengths

An overall comparison between the predictive power of water- and glucose-based PLS-R models indicated that the glucose-related regions G1 and G2 considerably outperformed the two water bands W1 and W2. The glucose regions yielded far lower prediction errors with NRMSEP values as low as 0.4% along with the utilization of only one PLS-R factor. This emphasizes the dominant presence of glucose-related spectral information in these two NIR regions at around 5900 cm${}^{-1}$ and 4400 cm${}^{-1}$. The only exception with poor predictive power was the 0.1 mm cell in region G1 due to the reasons described earlier. With regard to the pathlength, the 1 mm cell turned out to give the most accurate PLS-R models for both water and glucose related regions. This optical pathlength seemed to have a favorable ratio between transmitted and absorbed NIR light for quantitative analyses of aqueous glucose solutions and most probably for carbohydrate solutions in general.

## 4. Conclusions

The good predictive performance of the PLS-R models with the water-related regions W1 and W2 confirmed the well documented fact, in which sugars (in this case glucose) in aqueous solutions affect the water bands in the NIR region by disturbing the structure of the hydrogen bond network of liquid water [26,39]. On the other hand, considering the significantly higher predictive power of the PLS-R models based on the regions G1 and G2, it must be concluded that the regions associated with carbohydrate vibrations (i.e., C−H, O−H, C−O) are even better suited for highly accurate quantifications. These vibrations cause rather small bands in the NIR spectrum, thus derivative functions need to be applied in order to reveal the concentration dependent patterns.

The validity of the results obtained herein was further confirmed by the fact that multiple batches were used for the construction of the PLS-R calibration models. Moreover, independent test set samples were utilized in the process of PLS-R model validation. Therefore, a certain robustness of the constructed PLS-R models can be assumed.

This study demonstrated the superiority of characteristic glucose bands over the dominant and intense water bands in the NIR spectrum in terms of quantitative predictive power. Relative prediction errors lower than 1% were obtained while only one PLS-R factor was required. Further investigations need to be carried out in order to determine reliable values for the limit of detection (LOD) and the limit of quantification (LOQ) of both the water- and glucose-related regions. However, despite the promising predictive power of the glucose bands, it has to be noted that the sample matrix employed in the present study was rather simple. The establishment of reliable PLS-R models based on NIR data obtained from more complex matrices like body fluids such as blood or urine, or food products like beverages, is undoubtedly much more challenging. Nevertheless, the findings reported herein can support the selection of the most informative NIR regions for investigations of aqueous carbohydrate systems.

## Figures and Tables

**Table 1:** Details of spectral pre-treatments applied to each spectral region.

Cell	Region	Pre-Treatments
0.1 mm	W1	FFT-filter, SavGol smoothing (25 SP, second polynomial order), SNV
	W2	FFT-filter, SavGol smoothing (25 SP, second polynomial order), SNV
	G1	FFT-filter, SavGol smoothing (13 SP, second polynomial order), SNV, second SavGol derivative (15 SP, second polynomial order)
	G2	FFT-filter, second SavGol derivative (11 SP, second polynomial order)
1 mm	W1	SavGol smoothing (second polynomial order, 25 SP)
	G1	second SavGol derivative (19 SP, second polynomial order)
	G2	second SavGol derivative (25 SP, second polynomial order)
2 mm	W1	SavGol smoothing (25 SP, second polynomial order), SNV
	G1	second SavGol derivative (9 SP, second polynomial order)
	G2	second SavGol derivative (7 SP, second polynomial order)

SavGol—Savitzky-Golay; SP—smoothing points.

**Table 2:** Results of the PLS-R calibration and test set validation for all three cell pathlengths as well as for each spectral region.

Cell	Region	Type of	PLS-R	RMSEC	NRMSEC	RMSEP	NRMSEP	SEC	SEP	Bias	R^2^
		Validation	Factors	in mmol/L	in %	in mmol/L	in %	in mmol/L	in mmol/L	in mmol/L	
0.1 mm	W1	CAL	2	4.7	2.3	-	-	4.8	-	2.1 × 10${}^{-5}$	0.995
		TSV		-	-	7.6	3.8		5.6	5.6	0.98
	W2	CAL	2	2.7	1.4	-	-	2.8	-	5.5 × 10${}^{-5}$	0.998
		TSV		-	-	8.3	4.1	-	9.0	0.5	0.98
	G1	CAL	3	5.2	2.6	-	-	5.3	-	0	0.994
		TSV		-	-	22.6	11.3	-	23.1	−8.0	0.83
	G2	CAL	1	1.6	0.8	-	-	1.6	-	0	0.9994
		VAL		-	-	1.4	0.7	-	1.5	0.1	0.9993
1 mm	W1	CAL	2	2.1	1.0	-	-	2.1	-	2.3 × 10${}^{-5}$	0.9989
		TSV		-	-	3.2	1.6	-	3.2	1.1	0.997
	G1	CAL	1	1.2	0.6	-	-	1.3	-	0	0.9996
		TSV		-	-	1.8	0.9	-	1.9	0.4	0.9989
	G2	CAL	1	0.6	0.3	-	-	0.6	-	0	0.99992
		TSV		-	-	0.7	0.4	-	0.8	0.2	0.9998
2 mm	W1	CAL	3	4.0	2.0	-	-	4.1	-	1.5 × 10${}^{-5}$	0.996
		TSV		-	-	10.1	5.1	-	9.5	–5.3	0.97
	G1	CAL	1	1.1	0.6	-	-	1.1	-	0	0.9997
		TSV		-	-	1.5	0.8	-	1.6	–0.6	0.9992
	G2	CAL	1	3.1	1.6	-	-	3.2	-	0	0.998
		TSV		-	-	3.3	1.6	-	3.0	1.8	0.996

CAL—calibration; TSV—test set validation.

**Table 3:** Explained Y-variances for each spectral region and utilized cell. The type of validation (calibration or test set validation) and the number of PLS-R factors are specified. The values for the explained variances are given in %.

			PLS-R Factor			
Region	Cell	Type	1	2	3	4
**W1**	0.1 mm	CAL	95.5	99.5	99.8	99.9
		TSV	96.4	98.1	99.7	99.7
	1 mm	CAL	96.8	99.9	99.9	100
		TSV	97.7	99.7	99.8	99.5
	2 mm	CAL	48.1	98.8	99.6	99.9
		TSV	22.7	93.8	96.6	99.0
**W2**	0.1 mm	CAL	99.4	99.8	99.9	100
		TSV	95.3	97.7	97.9	99.2
	1 mm	CAL	-	-	-	-
		TSV	-	-	-	-
	2 mm	CAL	-	-	-	-
		TSV	-	-	-	-
**G1**	0.1 mm	CAL	96.8	99.2	99.4	99.6
		TSV	79.2	71.7	83.0	80.3
	1 mm	CAL	100	100	100	100
		TSV	99.9	99.9	99.9	99.9
	2 mm	CAL	100	100	100	100
		TSV	99.9	99.9	99.9	99.9
**G2**	0.1 mm	CAL	99.9	100	100	100
		TSV	99.9	99.9	99.9	99.9
	1 mm	CAL	100	100	100	100
		TSV	100	99.9	100	99.9
	2 mm	CAL	99.8	99.9	99.9	99.9
		TSV	99.6	99.6	99.4	99.6

CAL—calibration; TSV—test set validation.

## Abbreviations

The following abbreviations are used in this manuscript:

CAL	Calibration
FFT	Fast Fourier-Transform
FT-NIR	Fourier-Transform Near-Infrared
LOD	Limit of Detection
LOQ	Limit of Quantification
NIPALS	Nonlinear Iterative Partial Least Squares
NIR	Near-Infrared
NIRS	Near-Infrared Spectroscopy
NRMSE	Normalized Root Mean Square Error
NRMSEC	Normalized Root Mean Square Error of Calibration
NRMSEP	Normalized Root Mean Square Error of Prediction
PCA	Principal Component Analysis
PLS-R	Partial Least Squares Regression
RMSE	Root Mean Square Error
RMSEC	Root Mean Square Error of Calibration
RMSEP	Root Mean Square Error of Prediction
SavGol	Savitzky-Golay
SEC	Standard Error of Calibration
SEP	Standard Error of Prediction
SNV	Standard Normal Variate
SP	Smoothing Points
TSV	Test Set Validation
